# Including planetary health in continuing professional development

**DOI:** 10.4102/phcfm.v18i1.5524

**Published:** 2026-06-12

**Authors:** Robert J. Mash

**Affiliations:** 1 Division of Family Medicine and Primary Care, Faculty of Medicine and Health Sciences, Stellenbosch University, Cape Town, South Africa

When was the last time planetary health was addressed in your continuing professional development (CPD)? In a recent survey of the Primary Care and Family Medicine (PRIMAFAMED) network, 61% had never attended a CPD event on planetary health.^1^ It is very unlikely that undergraduate or pre-service training of the current African primary care workforce will have addressed planetary health. It is therefore important that we equip people through CPD, while also embedding planetary health in the training of the next generation. This editorial introduces a new special collection of CPD articles that will enable readers and CPD programmes to start addressing these issues.^2^

But, why is CPD on planetary health important? We know that there is a growing ecological crisis and that this has major health and social effects.^3^ The crisis is often framed as climate change, but also includes other issues such as loss of biodiversity (for example, the collapse of fisheries and associated livelihoods), pollution (for example, the inclusion of micro-plastics in our food chain) and changes in land use (for example, deforestation). This ecological crisis impacts health via several intermediaries, for example, changes in food production, changes in water quality and quantity, changes in exposure to infectious diseases and extreme weather events. These, in turn, lead to health effects across the burden of disease and social effects such as migration and conflict, which themselves have health consequences. The effects can be modulated by the wealth of a community, the use of technology or better governance, but most communities in Africa are vulnerable.

While there is no new climate-related disease, there will be changes in the morbidity and mortality facing clinicians and peaks in demand because of extreme events. I often conceptualise the issue as having three dimensions. The first is the health and social effects and how primary care providers can prepare themselves for this. The second is the effect of the same issues on the health facilities and services. For example, the president of Mozambique announced the loss of 472 primary care facilities to climate change.^4^ This illustrates why we need to create more resilient facilities and services. Finally, we need to reduce the environmental harm of the health sector. Health Care Without Harm have calculated that the health sector would be the fifth highest emitter of greenhouse gases if it were a country.^5^ In Africa, some countries already emit more per capita than high-income countries (e.g. South Africa),^6^ while many low- and middle-income countries have higher carbon intensity per dollar spent on healthcare than high-income countries.^7^ This means we need to be environmentally sustainable as we strengthen and develop our health services.

We asked members of the PRIMAFAMED network what topics should be included in CPD and then developed articles to address these topics.^1^ In this series, we have addressed the top five topics. Firstly, to understand in more detail how climate change leads to a variety of health and social effects. Secondly, to think about how you might develop the climate resilience of communities through a community-orientated primary care approach. Thirdly, to explore how you can assess and improve the climate resilience of your practice and measure its carbon footprint. Fourthly, to understand how you can contribute to emergency preparedness and disaster planning in your area. Lastly, to understand the clinical presentation and management of people with heat-related diseases. We will link these articles to multiple choice questions (MCQs) in eCPD^®^ (https://healthcare-ecpd.co.za) for individual use and to resources that can be downloaded for use in local CPD programmes.

In this same survey of the PRIMAFAMED network, we also identified several important clinical topics.^1^ These included respiratory diseases, diarrhoeal diseases, malnutrition, malaria and infectious diseases, mental health disorders, injuries and trauma, maternal and neonatal health, cardiovascular disease and diabetes. We plan to also develop CPD resources that can be embedded into presentations or CPD courses and briefly highlight the links to planetary health. These will be made available on the PRIMAFAMED website (https://primafamed.sun.ac.za).

**Funding statement:** The publication costs were paid by Stellenbosch University, Division of Family Medicine and Primary Care using a TEAM grant (ZA2022TEA526A103) from the Flemish Interuniversity Council (VLIR-UOS).
